# Report of a Case of Subcutaneous Emphysema Occurring in Connection with Pneumonitis, Ending in Recovery; with Remarks

**Published:** 1855-01

**Authors:** D. A. Legrand


### Art. II.—REPORT OF A CASE OF SUBCUTANEOUS EMPHYSEMA OCCURRING IN CONNECTION WITH PNEUMONITIS, ENDING IN RECOVERY; WITH REMARKS.*

BY D. A. LEGRAND.

I have thought that the readers of the *Gazette des Hôpitaux* will peruse with some interest the following case which has been already referred to in a communication made to the *Society of Practical Medicine* by my friend and colleague, D. Duhamel, (*Gazette des Hôpitaux*, August 28, 1852). The publication of the report has been delayed till now, from considerations of propriety incident to its having been submitted to the *Societé Académique de la Loire-inferieure.*

In the case communicated to the Society of Practical Medicine, the disease ended fatally, and it may be shown without difficulty that there is little room for the expectation of a favorable issue in the great majority of cases of this kind. The case, however, which I am about to report is not, in this respect, *unique*, and, hence, there is hope for researches as to the reasons for so great a difference in the ultimate consequences of lesions which, at first view, seem to be identical, or, at least, very analagous.

The following is an account of the case observed in my practice:—

On the 23d of May, 1852, I was called to attend a daughter of Mrs. Goumery, living in the *Rue de Verneuil.* The child, aged about five years, with a good constitution, having never suffered previously with disease, was affected with measles, of which the premonitory symptoms appeared on the 21st. The day after my first visit, a double pneumonia became developed, more extensive,

* Translated from the Gazette des Hôpitaux, for August 3, 1854, by Prof. A. Flint.

and its limits better defined on the right than on the left side. There were diminished sonoriety, especially behind, toward the base; complete absence of vesicular expansion in the inferior two-thirds of the right lung, and in the inferior half of the left lung; bronchial respiration marked on both sides; extreme oppression of breathing, 45 to 48 inspirations, and the pulse 170 per minute; cough incessant, and expectoration tinged with blood.

Notwithstanding the extent and the intensity of the internal inflammation, the eruption continued to develop itself regularly, and it is rare to see a case of measles more fully or better marked. Not deeming it proper, under the circumstances, to take blood, I prescribed an emetic mixture to be taken in doses repeated every two hours, night and day. After repeated acts of vomiting, and several dejections, tolerance was established, and this medication, together with two blisters on the scapula, was continued for six successive days. At the end of that period the fact of a distinct amelioration was confirmed by Dr. BLACHE, whom I called in consultation, at the desire of the mother.

A light chill occurring at the dressing of the blisters rekindled the inflammation which could no longer be combatted by emetics, in consequence of some symptoms of gastro-intestinal irritation. After two days, during which the treatment was altogether emollient and soothing (with the exception of two new blisters), I prescribed the kermes mineral, two doses of which, (given on the 4th of June), were followed by abundant evacuations from the stomach, relieving somewhat the lung, and on the 5th there occurred again a slight amendment of the condition of the patient. On the 6th, the oppression of breathing, which had diminished, returned, and the mother directed my attention to a swelling apparent over all the anterior and superior portion of the chest, the color of the skin remaining unchanged. In making, upon the swelled parts, slight pressure, pain was produced, and a *fine crepitation* was appreciable such as is felt when a portion of healthy pulmonary structure is compressed. The portion swelled was thus distended by gas, and was the seat of an *emphysema.*

What was the nature of the effused gas? Whence did it come? These were the two questions which suggested themselves. Did it escape from pulmonary vesicles ruptured by the efforts of coughing, or by the recent acts of vomiting? * But, then, it would at first have escaped into the cavity of the pleura, thence entering by some fissure to diffuse itself through the areolar tissue of the deep seated and superficial parts of the neck, and if so, there would have been collapse of the lung, with more marked symptoms of oppression amounting to suffocation, tending rapidly to a fatal result. Similar effects would have been produced, if the opening giving exit to the air was situated in one of the bronchial ramifications. The view, then, most probable was, that the walls either of the trachea or larynx had given way at some feeble points permitting the extravasation of air, and producing the phenomena which belong to the emphysematous goître described by pathologists. †

I entertained from the first, an unfavorable prognosis, the more because one of my colleagues had just lost one of his patients in consequence of a similar accident supervening on convalescence from typhoid fever. ‡

Appreciating my powerlessness before such a pathological manifestation, it was clear that there was nothing for me to do but to endeavor to quiet the cough which could not fail to increase the existing solution of continuity, wherever might be its seat; and I gave no other remedy than the syrup of poppy, at the same time keeping the lower part of the chest constantly covered with warm emollient cataplasms, and making over the emphysematous tumor (which was painful to the touch) soothing camphoreted embrocations.

---

* "However it is not very rare to see, in convulsive coughs, in certain attacks of asthma and during very violent muscular efforts, some bronchial cells to give way, the lung to become emphysematous, tumefaction from the presence of air to show itself about the clavicles, to extend over the neck, even to the summit of the chest."—Bégin, Dictionaire de Med. et de Chirurg. pratique, 1st edition, x. vii., p. 115, article Emphysème.

* "In most of the cases of the kind of those with which we are now occupied, the pulmonary tissue and the bronchial resist, but the walls of the trachea give way in their most feeble points and emphysema occurs in the cervical region."—Bégin, loc. cit.

* In this case, probably, the effused air escaping from some pulmonary vesicles ruptured, gained access into the pleural cavity, before being diffused in the subcutaneous areolar tissue.

The primitive tumor, which at first invaded the anterior and superior region of the chest, became extended over the entire right side of the body, so that crepitation was felt over almost the whole extent of the chest, and over the abdomen even to the neighborhood of the hip. Soon it was apparent on the left side of the neck, under the skin of the left cheek, the eye-lids, on the forehead, the eyebrow of the same side and at the root of the nose.

However, the cough and the oppression diminished daily, sleep returned, the appetite became developed, the effusion of air ceased to extend, and, at length, the child was fairly convalescent about the 15th of June. I discontinued my attendance on the 5th of July, and at that time for several days all traces of the emphysema had disappeared.

---

### REMARKS.

I have said, at the beginning, that the fact of cure in this case of emphysema is not *unique* in science. Thus, Dr. MAVEL, physician at Amber (*Peuy de Dôme*), communicated, the same year, an observation which presents a striking analogy to mine. (*Gaz. des Hôp.*, No. 133; 1852.) Since then, M. NATALIS GUILLOT, physician at Hospital NECKER, in a memoir inserted in the *Archives Génerales de Medicine*, 1853, vol. ii. p. 151,* reported a second instance of cure under circumstances nearly identical; I say *nearly* identical, for the case observed in the service of M. N. GUILLOT, being a child of eight months, the conditions were more unfavorable.† Afterward, Dr. Ch. ORAVAM, librarian of the Academy of Medicine, in a memoir on the same subject, inserted in the same collection, (January, 1854), has communicated an observation in which is presented a third instance of cure, and which bears still greater resemblance to the case which I have reported, save that M. ORAVAM, who called in consultation M. HENRI

---

* Observations d'emphysème siégeant sous la plèvre, dans le tissu cellulaire du mediastin, étendu jusqu'aux régions du con, du tronc, de la tête. et des membres, que l'ou pent àttribuer aux efforts de la toux chez les enfans.

* De la rupture pulmonaire chez les enfans et de l'emphysème général qui lui succède.

ROGER,* evacuated the air effused into the areolar tissue (in much greater quantity than in the case reported by me) by means of punctures made with a very fine trocher, whilst I relied upon the efforts of nature for its removal.

Here, then, inclusive of the case which I have now reported, are four cases of subcutaneous emphysema dependent on some lesion of the respiratory apparatus, *terminating in recovery;* but by the side of so small a number of favorable cases, M. ORAVAM opposes twenty-eight cases in which it may be said that the issue was fatal, although in three the fact of death was not positively ascertained, but is presumed to have occurred from its having been imminent when the cases were last seen.

It is important to inquire wherefore ended in recovery these four cases of a disease which appears to be generally fatal. *Dr. BEGIN who seems to be the first who has studied the subject (the article already cited was published in 1831) it appears to me has answered this question in the article from which I have borrowed two notes appended to this report. A cure takes place when the lesion which gives passage to the effused air is situated in the larynx or trachea, whilst in the greater number of instances, as fully established by M. NATALIS GUILLOT, who has brought to bear on this obscure point in pathology a flood of light, the solution of continuity giving passage to the air, is situated on the surface of the lung itself, becoming a powerful obstacle to the play of the organ; thence the air extends into the mediastinal areolar tissue, and, following the sheaths of the vessels, and the air tubes, where less resistance is met with, it gains the neck, which, apparently, in the large majority of cases (ROGER), is the first part where its presence is manifested.

---

* This physician has communicated to the medical society of the hospitals of Paris a memoir on the same subject (general emphysema in children) which has been inserted in the Journal des connaissances médicales, and reproduced in the Revue Medicale (Aug. 15, 1853). In this publication M. Roger establishes conclusively that the air effused into the areolar tissue can have no other origin than from a rupture in some portion of the respiratory organs, either the lungs or air tubes.

*Many distinguished authors, and among others M. M. Louis and Jackson have described subpleural emphysema. But much less is known of subcutaneous emphysema, due to the escape of air into the mediastinal areolar tissue, and thence into other parts.

As to the value of the explanation given by M. BEGIN of the favorable termination, independently of the authority of his name, it is confirmed by the case of a young girl, 15 years of age, (notwithstanding the general emphysema terminated fatally) in which there was ascertained to exist in the right ventricle of the larynx, a little below the vocal chord, a small round ulcer pierced in its centre by an opening of about the size of the head of a pin. * A lesion determined here by a pathological cause may be produced mechanically, in the same place, or in its vicinity. The different authors whom I have cited agree in admitting a mechanical agency and place in the first rank of agents the effects of coughing.

Whooping-cough is one of the most frequent causes of the affection to the consideration of which this paper is devoted, according to LAENNEC, and of which no one can doubt, after the following passage quoted from M. GUILLOT, who has seen "emphysema to occur, so to speak, under his eyes, by the continued agency of cough, causing air to be diffused beneath the integument of the whole body." The same practitioner thinks, as it seems to me correctly, that pulmonary emphysema so difficult of diagnosis when it is not complicated with subcutaneous emphysema, probably is not cured in cases the most favorable; so that, according to him, the phenomena of emphysema observed sufficiently often in adults, recognises a cause which may be traced to early infancy. I borrow of M. ORANAM a fact which proves the importance of a mechanical lesion as the cause of this affection ; it is, that in two cases of pneumonia the pulmonary rupture was recognized to exist on the healthy side.

But cough is not the sole cause of rupture of the pulmonary vesicles. Thus M. ROGER refers to a case communicated to the Academy of Medicine (27th Oct., 1846,) by Prof. VELPEAU, in behalf of M. VITALI, of general emphysema occurring (without an exterior lesion) in an infant, in consequence of an effort to disengage the arm from that of another infant. *Arch. Gén. de Méd.*, 1846; 4*e Sérıe*, x. xii. p. 372). He gives afterward an account of a very young child attacked with croup, tracheotomy having been

---

* This observation, cited by Dr. Roger, was published in the Gazette Médicale de Paris, 1840, x. viii. p. 698.

practiced, in whom infiltration of air appeared to take place from the borders of the wound. He appends the case of a child, aged nine years, attacked with hydrophobia, who presented, a short time before death, emphysema almost general. In the case of M. DUHAMEL the cough was quite insignificant.

As to therapeutical indications they are admirably portrayed in an excellent article published in this journal (Jan. 17, 1854) on the same subject by my colleague and friend, Dr. BOUCHAT. To that article I cannot do better than to refer my readers.

